# Ferroptosis and oral squamous cell carcinoma: connecting the dots to move forward

**DOI:** 10.3389/froh.2024.1461022

**Published:** 2024-09-04

**Authors:** Alessandro Antonelli, Anna Martina Battaglia, Alessandro Sacco, Lavinia Petriaggi, Emanuele Giorgio, Selene Barone, Flavia Biamonte, Amerigo Giudice

**Affiliations:** ^1^ Department of Health Science, School of Dentistry, “Magna Graecia” University of Catanzaro, Catanzaro, Italy; ^2^ Laboratory of Biochemistry and Cellular Biology, Department of Experimental and Clinical Medicine, “Magna Graecia” University of Catanzaro, Catanzaro, Italy

**Keywords:** oral squamous cell carcinoma, ferroptosis, autophagy, prognosis, signature, ferroptosis inducers

## Abstract

Oral squamous cell carcinoma (OSCC) is an aggressive disease whose incomplete biological comprehension contributes to the inappropriate clinical management and poor prognosis. Thus, the identification of new promising molecular targets to treat OSCC is of paramount importance. Ferroptosis is a regulated cell death caused by the iron-dependent accumulation of reactive oxygen species and the consequent oxidative damage of lipid membranes. Over the last five years, a growing number of studies has reported that OSCC is sensitive to ferroptosis induction and that ferroptosis inducers exert a remarkable antitumor effect in OSCC, even in those displaying low response to common approaches, such as chemotherapy and radiotherapy. In addition, as ferroptosis is considered an immunogenic cell death, it may modulate the immune response against OSCC. In this review, we summarize the so far identified ferroptosis regulatory mechanisms and prognostic models based on ferroptosis-related genes in OSCC. In addition, we discuss the perspective of inducing ferroptosis as a novel strategy to directly treat OSCC or, alternatively, to improve sensitivity to other approaches. Finally, we integrate data emerging from the research studies, reviewed here, through in silico analysis and we provide a novel personal perspective on the potential interconnection between ferroptosis and autophagy in OSCC.

## 1 Introduction

Oral squamous cell carcinoma (OSCC) is an aggressive disease and one of the most prevalent head and neck malignancy (1). The pathogenesis of OSCC is a multistep process characterized by the accumulation of genetic and epigenetic events that lead to the dysregulation of oncogenic signaling pathways, such as the epidermal grow factor receptor (EGFR) (2), PI3K/AKT/mTOR (3), Wnt/β-catenin (4), and JAK/STAT (5) as well as impairment of suppressor pathways, such as TP53/RB and p16/Cyclin D1/Rb (6, 7), overall involved in the control of cell growth, proliferation, differentiation, and cell death (8). In addition, the role of tumor microenvironment (TME) in shaping oral cancer progression is increasingly recognized. TME is often an inflamed ecosystem characterized by high levels of oxidative stress and hypoxia due to exposure to risk factors, such as microbial infections and tobacco smoke (9–11). Oral TME is also populated by diverse cell types such as immune cells and stromal cells which crosstalk through autocrine–paracrine signaling pathways, thus controlling the proliferation and metastasis of tumor cells as well as hindering effective drug diffusion and immune cell invasion (12, 13). OSCC clinical management is still a serious challenge. It requires aggressive multimodality approaches, including surgery followed by radiotherapy alone or with chemotherapy. In addition, although targeted therapies, such as cetuximab against EGFR, and the pembrolizumab- and nivolumab-based immunotherapy, have been approved by the Food and Drug Administration (FDA), the prognosis is poor, over half of OSCC patients experience locoregional recurrence or metastasis, and the 5-year survival rate is still less than 50% (1–17). Besides, clinical and histopathological factors used in clinical practice to predict the prognosis of patients with oral cancer are not satisfactory yet. The tumor-node-metastasis (TNM) staging system is the most widely used for risk stratification. However, patients with the same TNM stage may show significantly different clinical outcomes and responses to treatment (18). This suggests that the diversity within cancer extends beyond clinical and pathological aspects, but rather is fundamentally driven by a still incompletely defined complex array of biological alterations.

Over the past decade, the growing understanding of the molecular mechanisms underlying the regulated cell deaths (RCDs), in particular the so-called non-apoptotic RCDs, such as autophagic cell-death (ACD), ferroptosis, necroptosis, and pyroptosis, has shed light on new key features of oral carcinogenesis. Moreover, it provided novel therapeutic targets and strategies to hit OSCC where defective apoptosis pathways often tip the balance in favor of tumor progression (19). Autophagy is a dynamic process during which cells digest organelles and proteins to meet their metabolic needs and is tightly regulated by autophagy-related genes (ATG) (20, 21). Autophagy acts as a “double edged sword” in OSCC by promoting either tumor progression or tumor suppression (22). To make few examples, autophagy can favor the early steps of oral carcinogenesis by protecting OSCC cells from nutrients and oxygen depletion caused by insufficient vascularization (23); besides, autophagy inhibition can enhance cisplatin sensitivity in OSCC cell lines (24). Conversely, in other studies, it has been demonstrated activating autophagy, i.e., by inhibiting mTOR pathway, suppresses tumor activity in OSCC (25). To make thing more complex, autophagy has been found to play crucial roles in ferroptosis execution through several mechanisms, such as ferritin degradation (ferritinophagy), which leads to intracellular iron accumulation, and degradation of lipid droplets (lipophagy), which causes lipid peroxidation, and mitophagy. The autophagy cargo receptor NCOA4 binds to ferritin and deliver it into lysosomes where it is degraded. Ferritin phagocytosis promotes iron release and accumulation in cytoplasm and causes ferroptosis in tumor cells (26, 27). The autophagic degradation of lipid droplets causes the release and accumulation of free fatty acids, which in turn promote lipid peroxidation and subsequent ferroptosis (28). As such, ferroptosis is now described as an autophagic cell death (28, 29).

Ferroptosis is an oxidative type of RCD driven by the iron-dependent accumulation of reactive oxygen species (ROS), followed by peroxidation of lipid membrane and mitochondrial dysfunction (26, 30, 31). A number of studies demonstrate that ferroptosis occurs in OSCC and it is mainly associated with the impairment of System Xc−/GSH/GPX4 antioxidant pathway. Notably, interfering with this pathway or with intracellular iron and ROS levels, either through genetic modification or the administration of natural and chemical compounds, have shown a remarkable therapeutic potential (29, 32–38). Pyroptosis and necroptosis represents two additional targets for OSCC treatment: however, studies are limited and both the biological mechanisms and the implications of these RCDs in OSCC are still poorly understood (19). Pyroptosis is mediated by caspase activation through either canonical or non-canonical pathways (39). Necroptosis is initiated by tumor necrosis factor (TNF) signal and is regulated by a necrosome complex composed by receptor-interacting kinases 1 and 3 (RIPK1 and RIPK3) and Mixed lineage kinase domain-like protein MLKL (40). Both, pyroptosis and necroptosis induce a lytic cell death accompanied by the release of pro-inflammatory cytokines (i.e., interleukin-1β) and damage-associated molecular patterns (DAMPs) (41). Considering, though, that inflammation has the potential to increase the risk of tumorigenesis, promote metastasis, and cause treatment failure, it suggested that pyroptosis and necroptosis may play a role in OSCC development and progression (19).

Since its first steps, ferroptosis research in OSCC highlighted that the pronounced imbalance between oxidative stress and antioxidant defenses makes this tumor a breeding ground for ferroptosis onset. Oral precancer and cancer are characterized by heightened levels of ROS and reactive nitrogen species (RNS), not only due to the increased metabolism driven by abnormal cell growth, but also by the overexpression of enzymes like nitric oxide synthase (NOS), cyclooxygenase (COX), and lipoxygenase (LOX), which are significant sources of both ROS and RNS. Furthermore, environmental factors such as tobacco smoke, alcohol, and pathogens infections exacerbate the production and the adverse effects of ROS and RNS (9–11, 42). Originally, overwhelming literature has extensively documented the pivotal role of oxidative stress in both initiating and promoting the multistep process of oral carcinogenesis. In agreement with this assumption, treatment interventions, such as the use of antioxidants, and even lifestyle interventions, such as quitting smoking and reducing alcohol consumption, has shown the potential to mitigate OSCC risk and improve treatment outcomes. However, a different current of thought suggests that in certain contexts antioxidants can protect cancer cells from oxidative damage, thus inhibiting cell death and promoting the survival and growth of oral cancer cells (43). In this last scenario, the concept of ferroptosis has stressed the hypothesis that exasperate the production of free radicals, rather than attenuate it, might represent an alternative approach to kill oral cancer cells. Therefore, achieving a comprehensive and in-depth understanding of the role of ferroptosis in OSCC is crucial for effectively guiding its application in OSCC treatment.

In this review, we summarize the so far identified ferroptosis regulatory mechanisms and prognostic models based on ferroptosis-related genes (FRGs) in OSCC. We also discuss the perspective of inducing ferroptosis as a novel strategy for OSCC treatment by describing the ferroptosis-based therapeutic interventions currently used to kill OSCC cells or to improve sensitivity to other therapeutic approaches. Furthermore, we integrate data emerging from the research studies reviewed here and we provide a novel personal perspective on the potential interconnection between ferroptosis and autophagy in OSCC that could offer “food for thought” for further studies. Considering that squamous cell carcinoma (SCC) constitutes most of oral cancer cases, the present review focuses on oral SCC (OSCC) affecting tongue, mouth floor, gingiva, and buccal mucosa.

### 1.1 Ferroptosis: core regulatory mechanisms and morphological features

**Figure 1:**
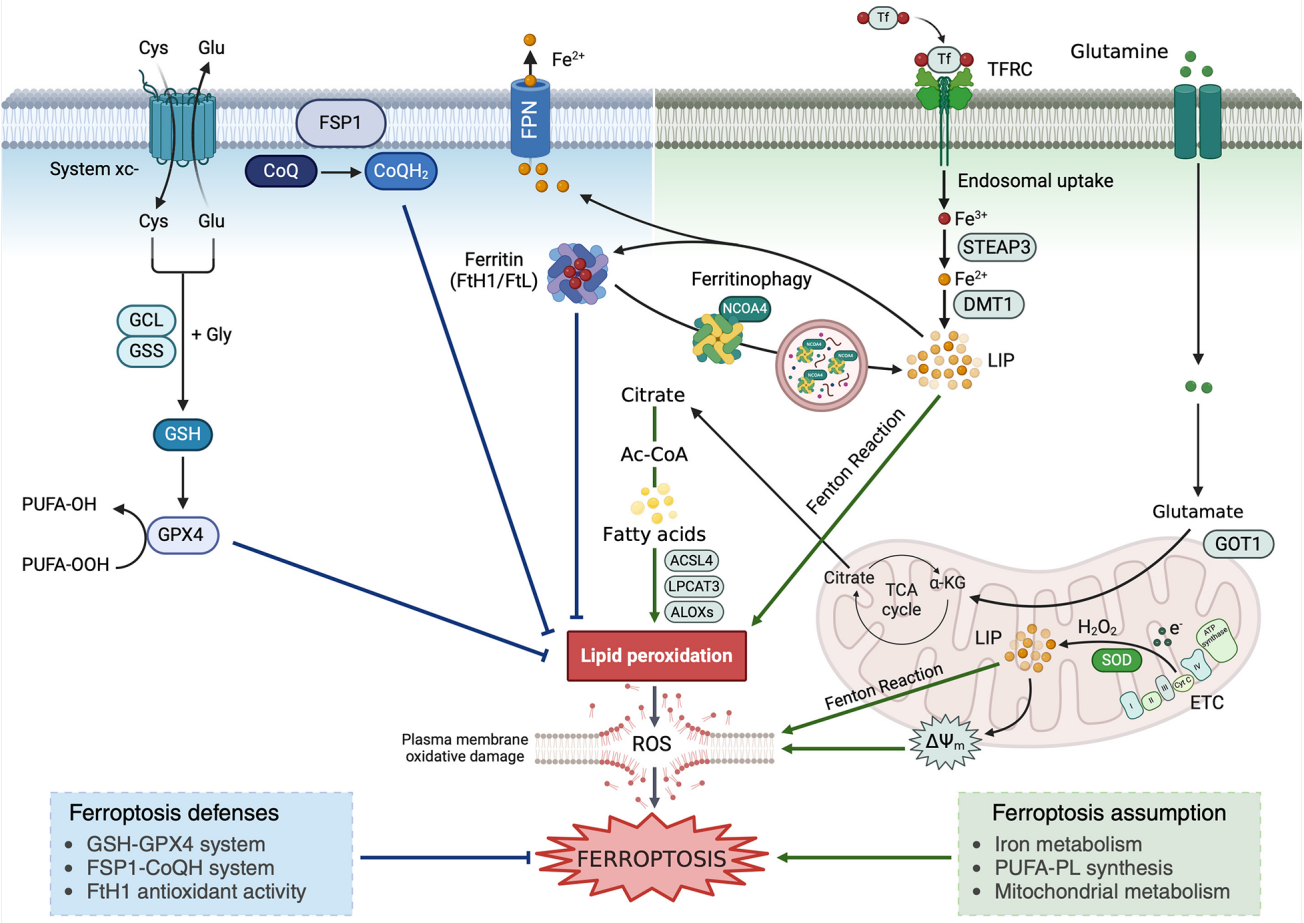
Ferroptosis occurrence relies on the antagonism between the promoting (right) and defense mechanisms (left). Graphic representation of the main ferroptosis-promoting events and ferroptosis defense mechanisms. Cys, cysteine; Glu, glutamate; Gly, glycine; GCL, glutamate cysteine ligase; GSS, glutathione synthetase; GSH, reduced glutathione; GPX4, glutathione peroxidase 4; PUFA, polyinsaturated fatty acids; CoQ, ubiquinone; CoQH2, ubiquinol; FTH1, ferritin heavy subunit 1; FTL, ferritin light subunit; FPN, ferroportin; FSP1, ferroptosis suppressor protein 1; NCOA4, nuclear receptor coactivator 4; DMT1, divalent metal trasposter 1; TFRC, transferrin receptor; Tf, transferrin; ALOX, arachidonate lipoxygenase; LIP, labile iron pool; TCA Cycle, tricarboxylic acids cycle; GOT1, glutamic-oxaloacetic transaminase 1; coA, coenzyme A; ETC, electron trasport chain; SOD, superoxide dismutase; ACSL4, acyl-coenzyme A (CoA) synthetase long-chain family member 4; LPCAT3, lysophosphatidylcholine acyltransferase 3; ΔΨm, mitochondrial membrane potential.

First discovered by Dixon et al. in 2012, ferroptosis is a new form of RCD both biochemically and morphologically distinct from other forms of cell death. Ferroptosis is driven by the iron-dependent peroxidation of membrane phospholipids (PLs) containing polyunsaturated fatty acids (PUFA-PLs). When lipid peroxidation exceeds the buffering capability of antioxidant defense systems, the lethal accumulation of lipid peroxides on cellular membranes, and the subsequent membrane rupture, lead to ferroptotic cell death (Figure 1) (44–46).

Iron is a fundamental micronutrient playing a key role in a variety of cellular biological processes such as cell cycle, oxygen transport, electron transport chain (ETC)-mediated production of ATP, and various reactions involving iron-containing proteins, heme-containing proteins, iron–sulfur (Fe–S) cluster proteins, and iron-containing enzymes (47). Under physiological conditions, cells incorporate iron as ferric Fe^3+^ complexed with transferrin (Tf) via transferrin receptor-1 (TfR1). Afterward, Fe^3+^ dissociates from Tf and is reduced to ferrous iron (Fe^2+^), which can be transported to the cytosol via the divalent metal transporter l (DMT1), stored by ferritin, and exported into extracellular space by ferroportin (FPN) (48). When the intracellular amount of Fe^2+^ overwhelms the storage capacity of ferritin, it accumulates within the cytoplasm as labile iron pool (LIP), which participates in the Fenton and reactions to generate the hydroxyl radicals (49, 50). If ROS are not properly buffered by the cellular antioxidant machinery, they drive the non-enzymatic autoxidation of PUFA-PL. Alternatively, Fe^3+^ may act as an essential cofactor for arachidonate lipoxygenase (ALOX) enzymes responsible for the enzymatic lipid peroxidation (26, 31, 50–53). Therefore, dysregulation of proteins involved in iron metabolism, including the iron uptake TfR1, the iron storage protein ferritin, and the iron efflux protein FPN, can promote or suppress ferroptosis, by modulating the amount of intracellular LIP (54, 55). To make an example, the autophagic degradation of ferritin mediated by the nuclear receptor coactivator 4 (NCOA4), a phenomenon known as ferritinophagy, releases free iron into the cytoplasm thus promoting ferroptosis (26, 54). Conversely, the blockade of NCOA4 decreases the level of the LIP and suppresses ferroptosis (56).

In contrast to monounsaturated fatty acids (MUFA), PUFA-PL are highly susceptible to peroxidation. Hence, their synthesis is a fundamental prerequisite for ferroptosis occurrence. PUFA-PL synthesis depends on the activity of two enzymes, acyl-coenzyme A (CoA) synthetase long-chain family member 4 (ACSL4) and lysophosphatidylcholine acyltransferase 3 (LPCAT3). ACSL4 catalyzes the ligation of free PUFAs with CoA to generate PUFA-CoAs, which are subsequently re-esterified and incorporated into PLs by LPCAT3 to form PUFA-PLs. As mentioned before, the peroxidation of PUFA-PLs, then, is mainly a non-enzymatic autoxidation mechanism driven by the iron-dependent generation of ROS via Fenton reactions or, alternatively, a series of enzymatic reactions mediated by ALOXs (57). Inhibition of PUFA-PL synthesis, via inactivation of ACSL4 and LPCAT3, or suppression of ALOXs functions can block or attenuate ferroptosis (58). In contrast, inactivation of enzymes involved in MUFA-PL synthesis, such as stearoyl CoA desaturase 1 (SCD1) and ACSL3, sensitizes cancer cells to ferroptosis (59). One of the final products of lipid peroxidation is malonyldialdehyde (MDA) that contains two aldehyde groups that can react with thiol and amine groups of proteins, lipids, amino sugars, and nitrogenous bases of nucleic acids. Besides, they modify physical properties of cell membranes by increasing their permeability. This causes changes in electric potentials on both sides of the membrane, resulting in loss of integration of both the intracellular and the plasmatic membranes and inhibition of activity of membrane enzymes and carrier proteins (60).

Ferroptosis defense mechanisms involve cellular antioxidant systems that directly neutralize lipid peroxides. Ferroptosis defense systems can be divided into GPX4-dependent and GPX4-independent arms (61). The SLC7A11–GSH–GPX4 axis constitutes the major cellular system defending against ferroptosis. GSH is a thiol-containing tripeptide derived from glycine, glutamate, and cysteine, with cysteine being the rate-limiting precursor. Cancer cells absorb cysteine mainly through the antiporter System xc–-mediated uptake of cystine, followed by cystine reduction to cysteine in the cytosol. SLC7A11 is the transporter subunit in System xc−. GSH is the cofactor of GPX4, an antioxidant enzyme able to detoxify lipid hydroperoxides converting them to lipid alcohols (62). Genetic ablation or pharmacological inhibition of GPX4 induces unchecked lipid peroxidation and triggers potent ferroptosis in many cancer cell types (29, 35). Among the GPX4-independent defense mechanisms, the ferroptosis suppressor protein 1 (FSP1), localized on the plasma membrane, functions as a NAD(P)H-dependent oxidoreductase capable of reducing ubiquinone (CoQ) to ubiquinol (CoQH2), which in turns traps lipid peroxyl radicals, thereby suppressing ferroptosis (63, 64).

Finally, mitochondria are key organelles in ferroptosis execution as they represent the major source of ROS. During the activity of the ETC complexes I and III, the electron leakage generates superoxide (O_2_^−^) that is converted to hydrogen peroxide (H_2_O_2_) by superoxide dismutase (2 HO_2_ → O_2_ + H_2_O_2_). H_2_O_2_ can, then, react with LIP via Fenton reactions to generate hydroxyl radicals, which subsequently drive PUFA-PL peroxidation (65).

**Figure 2:**
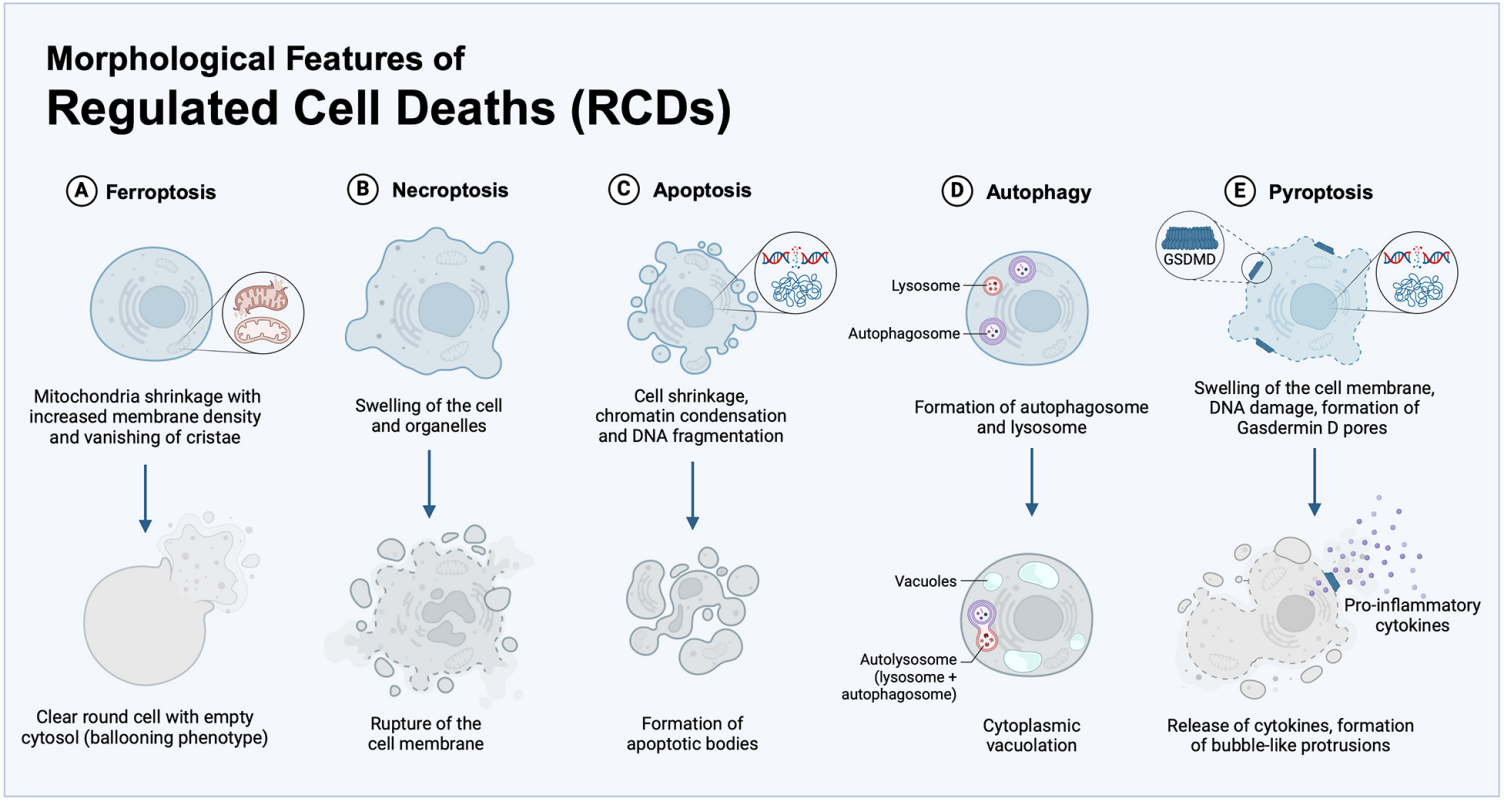
Morphological features of RCDs. Typical morphology of the main RCD processes, here including: **(A)** ferroptosis, identified by shrinkage of mitochondria with vanishing cristae and clear round cells with empty cytosol (ballooning phenotype); **(B)** necroptosis, characterized by membrane rupture and swelling of organelles, cellular collapse, and release of cellular contents; **(C)** apoptosis, defined by cell contraction, chromatin condensation, DNA fragmentation, and generation of apoptotic bodies; **(D)** autophagy, marked by the cytoplasmic vacuolation and autophagosome generation; **(E)** pyroptosis, that exhibits DNA damage and chromatin condensation, the formation of Gasdermin D (GSDMD) pores for secretion of inflammatory cytokines and bubble-like protrusions on the cellular membrane.

When observed through electron microscopy, ferroptosis presents with morphological features that make it unique compared to the other RCDs (Figure 2). Cells undergoing ferroptosis, indeed, do not show chromatin condensation, DNA fragmentation, apoptotic bodies or bubble-like protrusions as cells undergoing apoptosis and pyroptosis (66). Ferroptotic cells do not show organelle edema and content release with inflammatory response as cells undergoing necroptosis and pyroptosis (67). Ferroptotic cells appear as clear round cells with empty cytosol (the so-called ballooning phenotype) characterized by shrinkage of mitochondria with increased membrane density and vanishing mitochondrial cristae (26, 31). Ferroptotic cells may show cytoplasmic vacuolation and autophagosomes, which are typical characteristics of autophagy (68). However, the rapid vesicle turnover and the random distribution of vesicles within cells make the assessment of autophagosomes formation and degradation challenging (69).

### 1.2 Overview of ferroptosis in cancer

Since its discovery in 2012, ferroptosis has sparked great interest in the cancer research community. In particular, targeting ferroptosis has provided new therapeutic opportunities in treating cancers that are refractory to conventional therapies (45, 70–72). Ferroptosis is implicated in tumor biology in multiple ways. First, it makes a cancer cell nutrient addiction, i.e., iron addiction, a targetable vulnerability (26, 55, 73). Plus, it may exert anticancer activity by interacting with several tumor suppressors (74). For instance, the major tumor suppressor p53, among other functions, binds to and downregulates the cystine transporter solute carrier family 7 member 11 (SLC7A11), a component of the cystine/glutamate antiporter, thus causing reduced glutathione (GSH) depletion, the consequent glutathione peroxidase 4 (GPX4) dysfunction and ferroptosis in breast cancer cells (75). On the contrary, other evidence indicates that ferroptosis signaling pathways may promote tumor growth and progression. In this regard, it has been demonstrated that ferroptosis-mediated oxidative stress can activate two key transcription factors involved in cancer development and progression, namely nuclear erythroid factor 2-related factor (NRF2) and hypoxia inducible factor 1 alpha (HIF1-α). When chronically activated, NRF2 supports cancer cells proliferation, metabolic reprogramming, and resistance to therapy (76, 77). HIF-1α enhances the transcription of genes involved in cell survival, angiogenesis, and metastasis (78).

**Figure 3:**
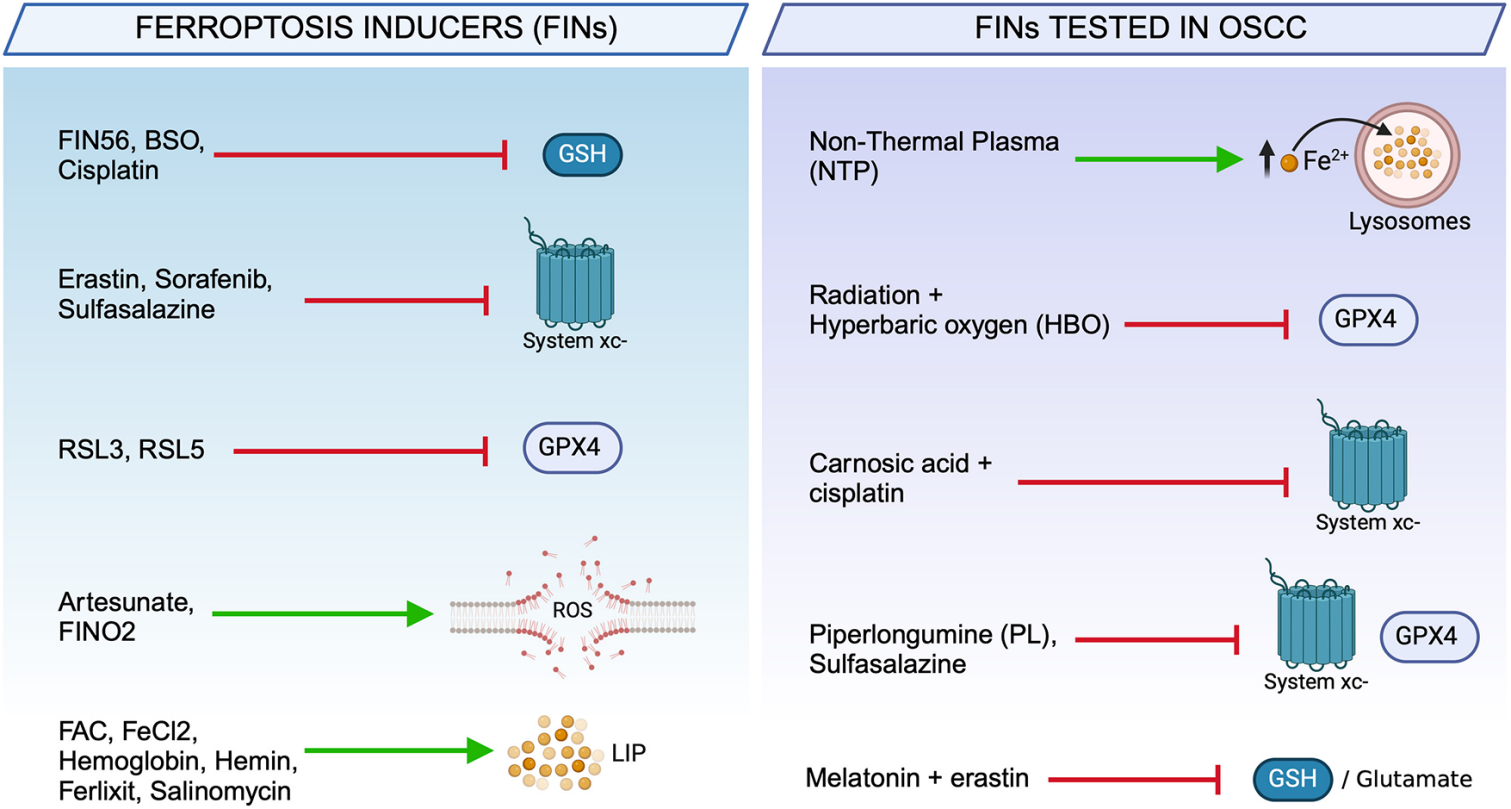
Chemical and natural compounds used to trigger ferroptosis. Ferroptosis inducers (FINs) used in cancer therapy classified according to their biochemical target (left). Ferroptosis-based therapeutic strategies used to trigger cell death in OSCC cells (right).

**Figure 4:**
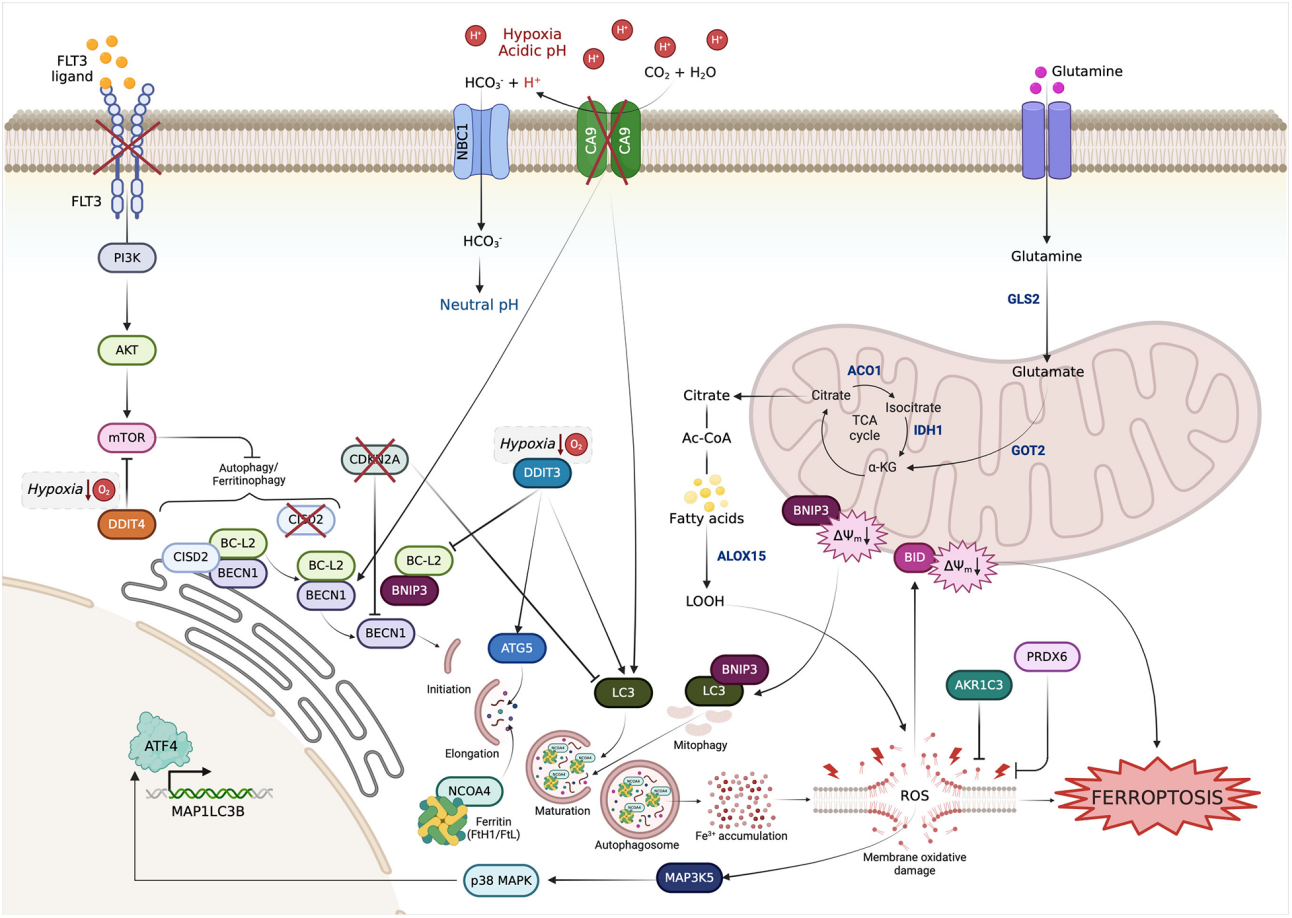
Interactions among the FRGs with prognostic value in OSCC based on autophagy. FRGs belonging to the prognostic models are involved in signaling pathways that converge on autophagy regulation.

As mentioned above, due to their metabolic requirements and oxidative burden, cancer cells may have a higher susceptibility to ferroptosis inducers (FINs). Over the last 5 years, several FINs have shown a great antitumor effect by targeting the tumor redox homeostasis, and their relative mechanisms of action have been elucidated. Today, the number of FINs is ever growing and can be classified as: (i) System Xc^−^ inhibitors (Erastin, Sulfasalazine, Sorafenib), (ii) GSH depleters (FIN56), (iii) GPX4 inhibitors (RSL3), and (iv) iron metabolism modulators (Figure 3) (46, 79). In addition, a series of drugs already approved by FDA in chemotherapy, radiotherapy, immunotherapy, and targeted therapies, have been found to act also via ferroptosis induction or synergistically with FINs in different cancer types (Figure 4) (80, 81). The poly (ADP-ribose) polymerase (PARP) inhibitor olaparib promotes ferroptosis by suppressing SLC7A11 and, thus, synergizes with RSL3 in BRCA-proficient ovarian cancer cells (82). GPX4 inhibitors enhance the sensitivity of resistant triple-negative breast cancer (TNBC) cells to the EGFR-tyrosine kinase inhibitor gefitinib (83). However, certain tumor cells inevitably adopt mechanisms of ferroptosis evasion, such as heightening the antioxidant capacity as an adaptive response to increased ROS levels or, alternatively, overexpressing iron efflux pumps to avoid LIP accumulation. In these cases, the combination therapy of FINs with other therapeutic approaches represents a promising strategy to tackle resistance and preventing tumor recurrence (74).

Ferroptosis plays a versatile and complex role of in the crosstalk between tumor cells and TME. Ferroptotic cells, indeed, release DAMPs that recruit and activate immune cells such as macrophages, initiating an antitumor immune response (84). Moreover, lipid peroxidation may also increase T cell-mediated cytotoxicity against tumor cells (85). Simultaneously, mediators released by immune cells also have a crucial impact on regulating the susceptibility of cancer cells to ferroptosis. Notably, the occurrence of ferroptosis in tumor infiltrating immune cells affect their survival and immunomodulatory function, ultimately reprograming tumor progression in the TME (86).

## 2 Literature search strategy and characteristics of the selected studies

To discover the eligible studies reporting the roles of ferroptosis in OSCC, the survey of the literature was conducted on 4 life-science databases: MEDLINE (PubMed), Scopus, Web of Science, and Google Scholar. The timeframe of searching was spanned from the inception of these databases to 2023. In each database, the searching strategy used the following keywords: “Ferroptosis”, “Oral squamous cell carcinoma”, “Oral cancer”, “Oral carcinoma”, and “OSCC”. Original research articles, i.e., *in vitro*, *in vivo*, and *in silico* studies were selected. Conversely, articles that were either unavailable in English language or unavailable as full text were excluded. The preliminary screening was assessed based on titles and the evaluation of abstracts, followed by the analysis of the entire texts. The key features of these studies, including the first author's name, the country where the studies were conducted, the publication year of the article, the type of study, materials and methods, and the main findings are summarized in Table 1. Briefly, the selected studies were conducted in 4 different countries (*n* = 14 in China, *n* = 1 in Japan, *n* = 1 in Taiwan, *n* = 1 in Japan and China, and *n* = 1 in Japan and Australia) and published between 2019 and 2023. The methodological approach of these studies was either experimental research (*n* = 11, 61%) or bioinformatics analysis (*n* = 7, 39%). Among the experimental research studies, *n* = 7 were based exclusively on *in vitro* assays in OSCC cells and *n* = 4 combined *in vitro* and *in vivo* assays, the latter performed with xenograft murine models or OSCC patients-derived tissue specimens. Most of the *in silico* studies used molecular and clinical data extracted from online dataset such as The Cancer Genome Atlas (TCGA), Gene Expression Omnibus (GEO), and International Cancer Genome Consortium (ICGC).

**Table 1:** Main characteristics of studies on ferroptosis and OSCC included in the review.

Ref.	Country	Type of study	Materials and methods^a^	Main finding
(87)	Japan/Australia	*In vitro*	OSCC cell lines	Identified non-thermal plasma as ferroptosis inducer
(88)	Japan/China	*In silico*	Gene expression analysis from TCGA and GEO	Identified a ferroptosis-based score, associated with OSCC TME composition, prognosis and treatment response
(89)	China	*In silico*	Gene expression analysis from TCGA	Constructed a 10-FRGS signature and relative nomogram associated with OSCC prognosis
(90)	Japan	*In vitro*	OSCC cell line	Identified miR-7-5p as regulator of IR resistance via ROS generation and ferroptosis
(29)	China	*In vitro* *In vivo*	OSCC cell lines Xenograft murine model	Identified circFNDC3B/miR-520d-5p/SLC7A11 axis inhibited GPX4 and SLC7A11 and increased ROS and Fe^2+^, attenuating ferroptosis
(91)	China	*In vitro*	OSCC cell lines	Identified carnosic acid as natural compound sensitizing OSCC cells to cisplatin by dysrupting Nrf2/HO-1/SystemXc^−^ pathway and causing ferroptosis in I
(34)	China	*In silico*	Network Pharmacology analysis and molecular docking	Revealed EGFR, PTGS2, and HIF1A proteins as possible direct targets of GSH potentially useful in OSCC treatment.
(92)	China	*In silico*	Gene expression analysis from TCGA	Constructed a FRncRNA-signature associated with OSCC prognosis
(93)	China	*In vitro* *In vivo*	OSCC cell lines OSCC human tissues	Revealed co-exposure to hyperbaric oxygen and x-ray radiation as potential approach to cause ferroptosis by regulating GPX4
(94)	China	*In silico*	Gene expression analysis from TCGA	Constructed an 8-FRlncRNAs signature associated with OSCC prognosis
(36)	China	*In vitro*	OSCC cell line	Identified miR-34c-3p as inhibitor of SLC7A11 expression thus promoting ferroptosis
(33)	China	*In vitro* *In vivo*	OSCC cell lines Xenograft murine model	Identified PER1 as a ferroptosis inducer through the degradation of HIF-1α
(95)	China	*In silico*	Gene expression analysis from TCGA	Constructed a 4-FRGs signature associated with prognosis, immune TME, and response to immunotherapy of OSCC
(37)	China	*In vitro* *In vivo*	OSCC cell lines Xenograft murine model	Identified AEBP1 as a negative regulator of ferroptosis in cisplatin resistant OSCC cells via JNK/p38/ERK pathway
(96)	China	*In silico*	Gene expression analysis from TCGA, GEO, and ICGC	Constructed a 9-FRGs signature associated with OSCC overall survival
(97)	China	*In vitro* *In vivo*	OSCC cell line Xenograft murine model	Identified a synergistic anticancer effect of melatonin and erastin by inducing ferroptosis
(97)	China	*In vitro*	OSCC cell lines	Identified the natural compound piperlongumine as ferroptosis inducer
(38)	China	*In vitro*	OSCC cell lines	Revealed CDH4 silencing as a novel approach to increase GSH consumption, GPX inactivity and ferroptosis

^a^ TCGA, the cancer genome atlas; GEO, gene expression omnibus; ICGC, International Cancer Genome Consortium.

## 3 Targets of ferroptosis in OSCC

Over the past five years, a growing number of *in vitro* and *in vivo* studies have demonstrated that ferroptosis opens a new avenue for OSCC treatment, essentially by interfering with iron metabolism and redox homeostasis of OSCC cells. Moreover, *in silico* analysis have led to the development of different prognostic models and/or scores based on ferroptosis related genes (FRGs) that hold the promise to direct personalized treatments in the future.

### 3.1 Core regulatory mechanisms of ferroptosis in OSCC

The research landscape of ferroptosis and its implication in OSCC moved its first steps around five years ago. Knowledge on the core regulatory mechanisms driving ferroptosis in OSCC is summarized in Table 2. In the study by Zhou, Q. et al., it has been demonstrated that treatment with the ferroptosis inducer (FIN) sulfasalazine (SSZ) causes the suppression of the transcriptional factor adipocyte enhancer-binding protein 1 (AEBP1) and thus the downregulation of its target genes *GPX4* and *SLC7A11.* Consequently, OSCC cells are unable to buffer lipid peroxides caused by the accumulation of intracellular free iron and ultimately undergo ferroptosis (37). According to Yang, Y. et al., the forced overexpression of *PER1* causes ferroptosis in OSCC cells (33). *PER1* is a core circadian clock gene normally expressed at low levels in OSCC tissues compared to adjacent healthy counterparts. When overexpressed in OSCC cells, by using expression vector transduction, it binds to and degrade HIF-1*α* (33) that has been recently considered a ferroptosis suppressor in several *in vivo* tumor models (98). HIF-1*α*, indeed, may inhibit ferroptosis by promoting the lactate production and TME acidification, which is associated with ferroptosis repression, or may enhance the transcription of the glutamate transporter SLC1A1, thus promoting cystine uptake, GSH synthesis and ferroptosis resistance (99). In OSCC, Yang, Y. et al. demonstrated that PER1 overexpression causes the degradation of HIF-1*α*, which in turn, is associated with reduced expression of SLC7A11, the consequent inhibition of the System xc^−^/GSH/GPX4 axis, the accumulation of ROS, and ferroptotic cell death (33). R-cadherin (CDH4) is a cell adhesion protein belonging to the cadherins family. Xie, J. et al. found that CDH4 is commonly overexpressed in OSCC, where it participates in epithelial-to-mesenchymal transition (EMT), invasion, and metastasis (38). Notably, CDH4 knockdown raises the consumption rate of GSH, thus causing the suppression of GPX4 activity, the accumulation of lipid peroxides, and ferroptosis (38). In support of the key role of GSH in ferroptosis occurrence in OSCC cells, a complex network pharmacology analysis, recently demonstrated that targeting GSH dramatically reduces OSCC survival by causing the dysregulation of 14 ferroptosis target molecules, namely EGFR, PTGS2, HIF-1α, VEGFA, TFRC, SLC2A1, CAV1, CDKN2A, SLC3A2, IFNG, NOX4, DDIT4, CA9, and DUSP1. Among these, the molecular docking analysis highlighted that GSH shows a strong binding affinity to EGFR, PTGS2, and HIF-1α (34). The main role of HIF-1α as a ferroptosis repressor has been described above. EGFR regulates ferroptosis by interacting with mTOR and MAPK signaling pathways in ovarian and lung cancer as well as hepatocellular carcinoma and glioblastoma (100–103). Notably, the overexpression of EGFR and the downstream recruitment and activation of oncogenic molecules, including MAPK, PI3 K/AKT, and JAK/STAT3, is one of the principal events involved in the control of differentiation, proliferation, angiogenesis, and metastasis of OSCC (104, 105). The prostaglandin-endoperoxide synthase (PTGS2) encodes for the cyclooxygenase-2 (COX-2) enzyme devoted to the biosynthesis of endoperoxides, such as prostaglandins (PGs), in response to a variety of inflammatory stimuli (106). In oral cancer, COX-2 enzyme regulates the biosynthesis of prostaglandin E2 (PGE2), which in turn induces the vascular endothelial growth factor (VEGF) expression in endothelial cells and angiogenesis (107); besides, high expression levels of COX2 are associated with lymph node metastasis (108–110). Since endogenous endoperoxides, such as PTGS2, have been found as natural triggers for ferroptosis, PTGS2 is now considered a biomarker of ferroptosis in several cancer types, including colorectal cancer and gastric cancer (111, 112). In OSCC, the relationship among GSH, HIF-1α, EGFR, PTGS2, and ferroptosis is still limited to a molecular docking analysis and needs to be further investigated in future studies.

**Table 2:** Regulatory mechanisms of ferroptosis in OSCC.

Trigger	Targets	Regulatory mechanisms	Ref.
Sulfasalazine (SSZ)	GPX4 and SLC7A11	Suppression of the transcriptional factor adipocyte enhancer-binding protein 1 (AEBP1)	(37)
Overexpression of PER1	System Xc^−^/GSH/GPX4	Proteasomal degradation of HIF-1α	(33)
CDH4 knockdown	GPX4	Increase the consumption rate of GSH	(38)
GSH inhibition	EGFR, PTGS2, HIF-1α	Unclear	(34)
miR-34c-3p	SLC7A11	Reduction of cysteine intake, impairing GSH production and GPX4 antioxidant activity	(36)
circFNDC3B	SLC7A11	Molecular sponge for miR-520d-5p	(29)

### 3.2 Non-coding RNA modulate ferroptosis in OSCC

Non-coding RNAs (NcRNAs) are a group of non-coding transcripts with limited protein-coding potential that regulate many cell life activities by controlling gene expression and protein synthesis. Three major classes of functional ncRNAs are (i) short microRNAs (miRNAs), (ii) long ncRNAs (lncRNAs), and (iii) circular RNAs (circRNAs) (113). NcRNAs are frequently deregulated in cancer and are widely considered biomarkers in both tumor diagnosis and prognosis. In addition, ncRNAs are tightly involved in the pathogenesis of cancer by affecting tumor cell proliferation, tumor metabolism, angiogenesis, metastasis, and drug resistance. Thus, targeting ncRNAs represents a promising therapeutic option to treat cancer and improve patients’ outcome (52, 114–118). As researchers have deepened their interest in ferroptosis, a growing number of non-coding (nc) RNAs has been proven to regulate, either positively or negatively, ferroptosis or to impact the propensity of tumor cells to undergo ferroptosis (119, 120).

In OSCC, Sun, K. et al., found that miR-34c-3p, directly targets *SLC7A11* and reduces cysteine intake, thus impairing GSH production and GPX4 antioxidant activity. Consequently, OSCC cells undergo ferroptosis (36). Based on this study, miR-34c may function as a tumor suppressor in OSCC. miR-34c has previously been counted among the microRNAs involved in the regulation of head and neck cancer (HNSCC). Several studies report that miR-34c exerts a suppressive function on the growth and invasiveness of HNSCC and that low levels of miR-34c correlate with worse overall survival (OS) in patients with HNSCC (121, 122). Other studies, instead, show that miR-34c is upregulated in HNSCC tumor tissue compared to neighboring healthy tissues and that overexpression of miR-34c promotes the proliferation, migration, and invasion of OSCC cells *in vitro* (123, 124).

In addition to direct regulation of ferroptotic key players, miRNAs may indirectly regulate this cell death process via interaction with other ncRNAs, such as lncRNAs and circRNAs. CircRNAs are single-stranded ncRNAs that form circular conformations through non-canonical splicing or back-splicing events (125). Many circRNAs can act as microRNA sponges to relieve the inhibitory effect of miRNAs on their target genes and increase the expression level of corresponding genes (126). In the study of OSCC, Yang, J. et al. demonstrated that circFNDC3B acts as a molecular sponge for miR-520d-5p and, thus, relieves its inhibitory effect on *SLC7A11* target gene. As a result, *SLC7A11* is overexpressed. Consistently with these findings, Yang, J et al. observed that high levels of circFNDC3B correlate with a low susceptibility to ferroptosis of OSCC cells (29). Consistently, Chen et al. previously found that circFNDC3B is highly expressed in OSCC tissues and that circFNDC3B promotes proliferation, migration, and invasion of OSCC cells by targeting miR-1322/MED1 axis (127). Notably, these two studies agree with the cancer-promoting role of circFNDC3B in OSCC and propose two different mechanisms of action that need to be further investigated.

### 3.3 Ferroptosis and prognosis in OSCC

During the last decades, many efforts have been made to identify markers potentially correlated with OSCC outcome. As a result, several factors ranging from clinical and histopathological features to genetic and molecular alterations have been described. However, their use is still limited and only few of them are applied in the routine clinical practice (128).

Among the clinicopathological markers, tumor staging, angiogenesis, tumor budding, and perineural invasion are the most described. Tumor staging is typically correlated with prognosis. In OSCC, clinical stages II-III-IV significantly correlate with a worse survival. Indeed, while the 5-year survival rate for stage I oral cancer patients is around 80%, it breaks down to 53% for those with stage IV disease (129). Angiogenesis, quantified as number of blood vessels present in specific areas of the tumor, is significantly associated with poor prognosis, as it correlates with tumor size, nodal metastasis and relapse (130). Tumor budding is present in around 35% OSCC and, when present, correlates shorter overall survival and lymph node metastasis (131, 132). Perineural invasion (PNI), generally detected in around 18% of OSCC patients, correlates with poor prognosis (133, 134). More recently, specifically after the approval of pembrolizumab and nivolumab for treating recurrent or metastatic oral cancer, the overall assessment of tumor-infiltrating immune cells has emerged as a hot target for the prognostication of OSCC. The presence of tumor-infiltrating lymphocytes (TILs) in the invasive front of oral cancer has been reported as a promising biomarker for a better prognosis (135, 136). Hadler-Olsen E and Wirsing AM indicated that CD163^+^ tumor-associated macrophages (TAMs) and CD57^+^ natural killer (NK) cells were the most promising predictors of a better survival in OSCC patients (137). The potential role of CD163^+^ TAMs and CD57+ NK cells in predicting OSCC prognosis was also found by Huang, Z. et al., together with CD8^+^ TILs, CD45RO^+^ TILs and CD68^+^ TAMs (138).

Among the biological markers used to further assess the prognosis of OSCC patients, HPV infection represents the main predictor of a better prognosis in terms of a higher overall survival rate, a better treatment response, a reduced disease progression risk and recurrence. As such, HPV protein p16 is one of the most investigated prognostic biomarkers of OSCC (139, 140). Additional biomarkers bring insights into the identification of more aggressive OSCC, which may benefit from more specific therapeutic approaches, although most of them do not show a clinical utility (128). To make an example, FAM3C (the family with sequence similarity 3 member C) is upregulated in OSCC compared to healthy mucosa and epithelial dysplasia, and patients with a higher FAM3C expression are more likely to have a poor prognosis. In addition, the expression of FAM3C correlates with the expression of immune checkpoints such as PD-L1, VISTA, and B7-H4, EMT marker Slug, and the cancer stem cells (CSC) markers SOX2 and ALDH1 (141).

Currently, data supporting the relationship between ferroptosis and prognosis in OSCC are mostly based on *in silico* analyses. During the last 4 years, five different research groups, drawing from The Cancer Genome Atlas (TCGA) database, found that numerous ferroptosis-related genes (FRGs) and ferroptosis-related lncRNAs (frlncRNAs) were differentially expressed in OSCC tissues compared to healthy mucosa (89, 92, 94–96), thus further supporting the involvement of ferroptosis in the molecular pathogenesis of OSCC. Then, each of these five research groups established a distinct risk model, based on the expression of FRGs or frlncRNAs, able to cluster OSCC patients in “high risk” and “low risk” groups characterized respectively by a bad and a good prognosis, in terms of overall survival (OS) (Table 3). First, Li, H. et al., constructed a prognostic signature composed by 10 FRGs, namely Autophagy Related 5 (*ATG5*), BH3 Interacting Domain Death Agonist (*BID*), Aconitase 1 (*ACO1*), Glutamic-Oxaloacetic Transaminase 1 (*GOT1*), Aldo-Keto Reductase Family 1 Member C3 (*AKR1C*3), Glutaminase 2 (*GLS2*), Arachidonate 15-Lipoxygenase (*ALOX15*), Synthesis Of Cytochrome C Oxidase 2 (*SCO2*), Microtubule Associated Protein 1 Light Chain 3 Alpha (*MAP1LC3A*), Mitogen-Activated Protein Kinase Kinase Kinase 5 (*MAP3K5*) (89). Later, Fan et al. identified nine prognostic FRGs; three of them, *ALOX15*, *ATG5* and *MAP1LC3A,* were in common with the results by Li, H. et al. (89), the other 6 include CDGSH Iron Sulfur Domain 2 (*CISD2*), DNA Damage Inducible Transcript 4 (*DDIT4*), Carbonic Anhydrase 9 (*CA9*), Beclin 1 (*BECN1*), BCL2 Interacting Protein 3 (*BNIP3*), and Peroxiredoxin 5 (*PRDX5*) (96). Then, Yin, G. et al., found 5 FRGs with prognostic value for OSCC patients: the above mentioned *BNIP3*, together with Ferritin Heavy Chain 1 (*FTH1*), Fms Related Receptor Tyrosine Kinase 3 (*FLT3*), Cyclin Dependent Kinase Inhibitor 2A (*CDKN2A*), and DNA Damage Inducible Transcript 3 (*DDIT3*) (95). Two further prognostic models for OSCC patients were constructed based on frlncRNAs. Some of them, namely AC099850.3, AC090246.1, AL512274.1, and Myocardial Infarction Associated Transcript (MIAT) were common between the two models (92, 94). The prognostic model developed by Qiu, L. et al., also included Firre Intergenic Repeating RNA Element (FIRRE), Long Intergenic Non-Protein Coding RNA 1305 (LINC01305), AC079921.2, and Long Intergenic Non-Protein Coding RNA 524 (LINC00524) (94) while that of Li, T. et al., also contained ALMS1 Intronic Transcript 1 (ALMS1-IT1), AC021087.4, and HOTARM1 as risk factors and STARD4 Antisense RNA 1 (STARD4-AS1) as protection factor (92). To date, these data show a promising link between ferroptosis and prognosis of OSCC. In future studies, the predictive value of these prognostic models needs to be verified by additional clinical data and the inherent mechanism of these FRGs in OSCC needs to be unveiled.

**Table 3:** List of FRGs with prognostic value in OSCC.

Gene	Function (gene cards)	Function in ferroptosis (FerrDb)	Protective/risk factor	Expression: high risk vs. low risk	Ref.
*ATG5*	Autophagy protein 5; Involved in autophagic vesicle formation.	Driver	Risk	UP	(89, 96)
*BID*	BH3-interacting domain death agonist; The major proteolytic product p15 BID allows the release of cytochrome c.	Driver	Risk	UP	(89)
*ACO1*	Cytoplasmic aconitate hydratase; Iron sensor. Binds a 4Fe-4S cluster and functions as aconitase when cellular iron levels are high.	Driver	Risk	UP	(89)
*GOT1*	Aspartate aminotransferase, cytoplasmic; Biosynthesis of L-glutamate from L-aspartate or L- cysteine.	Uncertain	Risk	UP	(89)
*AKR1C3*	Aldo-keto reductase family 1 member C3; Catalyzes the conversion of aldehydes and ketones to alcohols.	Suppressor	Risk	UP	(89)
*GLS2*	Glutaminase liver isoform, mitochondrial; Plays an important role in the regulation of glutamine catabolism.	Driver	Risk	DOWN	(89)
*ALOX15*	Arachidonate 15-lipoxygenase; Non-heme iron-containing dioxygenase that catalyzes the stereo-specific peroxidation of free and esterified polyunsaturated fatty acids generating a spectrum of bioactive lipid mediators.	Driver	Risk	DOWN	(89, 96)
*SCO2*	Protein SCO2 homolog, mitochondrial; Acts as a copper chaperone, transporting copper to the Cu(A) site on the cytochrome c oxidase subunit II (COX2).	Uncertain	Protective	UP	(89)
*MAP1LC3A*	Microtubule-associated proteins 1A/1B light chain 3A; Ubiquitin-like modifier involved in formation of autophagosomal vacuoles (autophagosomes).	Driver	Protective	DOWN	(89, 96)
*MAP3K5*	Mitogen-activated protein kinase kinase kinase 5; Serine/threonine kinase which acts as an essential component of the MAP kinase signal transduction pathway.	Uncertain	Protective	UP	(95)
*FTH1*	Ferritin heavy chain; Stores iron in a soluble, non-toxic, readily available form.	Suppressor	Risk	UP	(95)
*FLT3*	Receptor-type tyrosine-protein kinase FLT3; Tyrosine-protein kinase that acts as cell-surface receptor for the cytokine FLT3LG and regulates differentiation, proliferation, and survival of hematopoietic progenitor cells and of dendritic cells.	Driver	Protective	DOWN	(95)
*CDKN2A*	Cyclin-dependent kinase inhibitor 2A; Acts as a negative regulator of the proliferation of normal cells by interacting strongly with CDK4 and CDK6.	Driver	Protective	DOWN	(95)
*DDIT3*	DNA damage-inducible transcript 3 protein; Multifunctional transcription factor in ER stress response.	Uncertain	Risk	UP	(95)
*CISD2*	CDGSH iron-sulfur domain-containing protein 2; Regulator of autophagy that contributes to antagonize BECN1-mediated cellular autophagy at the endoplasmic reticulum.	Suppressor	Risk	UP	(96)
*DDIT4*	DNA damage-inducible transcript 4 protein; Regulates cell growth, proliferation, and survival via inhibition of the activity of the mammalian target of rapamycin complex 1 (mTORC1).	Uncertain	Risk	UP	(96)
*CA9*	Carbonic anhydrase 9; Reversible hydration of carbon dioxide. Participates in pH regulation.	Suppressor	Risk	UP	(96)
*BECN1*	Beclin-1; Plays a central role in autophagy.	Driver	Risk	UP	(96)
*PRDX6*	Peroxiredoxin-6; Thiol-specific peroxidase that catalyzes the reduction of hydrogen peroxide and organic hydroperoxides to water and alcohols, respectively.	Suppressor	Risk	UP	(96)

Detailed description of the FRGs according to their role in ferroptosis and their protective/risk effect in OSCC prognosis based on their relative prognostic models.

### 3.4 Ferroptosis-related prognostic signatures and oral tumor immunity

Due to environmental pressures, such as alteration of microbiome, HPV infection, and stromal remodeling, OSCC is characterized by extensive infiltration of immune cells, including tumor-infiltrating lymphocytes (TILs) [T cells, B cells and natural killer (NK) cells] and myeloid lineage cells (macrophages, neutrophils, dendritic cells, and myeloid-derived suppressor cells) (1, 142). However, OSCC shows the ability to evade immune surveillance through a variety of strategies, some of them specific for OSCC and others shared with other solid tumors (1). First, tobacco smoke and alcohol promote a high mutational rate in human leukocyte antigen (HLA) genes that are critical for OSCC to evade immune surveillance (143). OSCC cells can directly enhance the expression of inhibitory molecules such as (i) programmed death ligand-1 (PD-L1), which interacts with programmed death-1 (PD-1) on T cells to suppress their activity, or (ii) cytotoxic T-lymphocyte-associated antigen-4 (CTLA-4), which interacts with CD80/86 on antigen-presenting cells (APCs) to block the differentiation of naïve T cells (144–147). Alternatively, OSCC cells can inhibit both innate and adaptive immune response by releasing immune-inhibitory cytokines, such as tumor growth factor-β (TGF-β) and interleukin-6 (IL-6), or suppressing the production of immune-activating cytokines, such as interleukin-2 (IL-2) (148, 149).

Like for other forms of RCD, ferroptosis plays critical roles in the regulation of antitumor immunity (150). First, ferroptosis can be considered an immunogenic cell death (ICD) as cells undergoing ferroptosis often release damage-associated molecular patterns (DAMPs) that modulate cellular immune response (151). Cancer cells dying from ferroptosis may release high-mobility group box 1 (HMGB1) and adenosine triphosphate (ATP), which in turn stimulate the macrophage-mediated production of tumor necrosis factor α (TNFα), thus causing inflammation (152). Pancreatic cancer cells undergoing ferroptosis release mutant *KRAS*^G12D^ proteins that are uptaken by macrophages, which in turn respond by adopting a pro-tumorigenic M2 phenotype (153). Immune cells resident within the TME, from their side, can undergo ferroptosis thus affecting the antitumor immune response. For instance, inducing ferroptosis in Tregs, by suppressing GPX4, leads to the production of the proinflammatory interleukin-1β (IL-1β), which in turn activates T helper 17 (T_H_17) response and enhances antitumor immunity (154). Alternatively, immune cells resident within the TME trigger ferroptosis in tumor cells. Interferon gamma (IFNγ), released by CD8^+^ T cells, downregulates the expression of the two subunits of the System Xc^−^, SLC3A2 and SLC7A11, thus promoting lipid peroxidation and ferroptosis in ovarian cancer and melanoma cells (85).

In the study of oral cancer Gu, W. et al., highlighted that OSCC tissues characterized by high levels of ferroptosis driver genes showed a TME that they defined as “immune-inflamed”, since characterized by high expression levels of immune inhibitory molecules, such as PD-L1 and CTLA-4, and high infiltration of immune cells. Notably, this group of OSCC patients exhibited clinical features associated with a better prognosis, i.e., low-grade, early-stage tumors, no lymph node metastasis, and good response to immune checkpoints (ICIs)-based immunotherapy (88). Besides, it is worth noting that each of the five signatures described above, independently from their composition, was characterized by FRGs or frncRNAs functionally involved, among others, in the modulation of immune response according to the Kyoto Encyclopedia of Genes and Genomes (KEGG) pathway enrichment analysis. Indeed, a further analysis of the TME of OSCC patients belonging to the “high-risk” group with poor prognosis highlighted that these patients were characterized by a TME poor of APCs and relative functions, especially immature dendritic cells (iDCs and B cells), and by a low percentage of immune effector cells including CD8^+^T cells and NK cells (89, 92, 94–96). Considering that, when present, iDCs and B cells favor the immune activity against OSCC (85) and that, CD8^+^T and NK cells kill tumor cells and, thus, prevent OSCC growth and metastasis by remodeling of the oral TME via IFN-γ and TNF-α (155), the authors concluded that the bad prognosis of the “high-risk” OSCC patients was, in some way, associated with the impairment of the antitumor immune response.

Overall, these data highlight that ferroptosis-based multigene signatures may provide a valid tool to predict both the immune status and prognosis of OSCC patients. However, so far, these data lack experimental validation and, necessarily, need a complete molecular characterization before being used as prognostic biomarkers or targets for novel therapies.

### 3.5 Targeting ferroptosis to treat OSCC

OSCC requires a multidisciplinary approach including surgical intervention followed, if necessary, by postoperative radiation or chemotherapy. In most cases, surgery is the first-line treatment for oral carcinomas. In advanced cases, postoperative radiation, chemoradiation, oncogene-targeted therapy, and immunotherapy may be administered (1). However, aggressive OSCC have a poor prognosis, with limited improvements in survival over many decades (18, 136, 156).

Given the role of oxidative stress in carcinogenesis of OSCC, both small molecules and natural compounds targeting the redox balance have become appealing targets for intervention in this field. Treatment with polyenolpyrrole auxarconjugatin B causes DNA damage and apoptosis via generation of intracellular ROS in oral squamous cell carcinoma xenografts (157). Cisplatin-resistant oral cancer cells exposed to curcumin nanoparticles show increased production of ROS in association with increased levels of apoptotic proteins, such as caspase 9, cytochrome c, and apoptotic protease activating factor 1 (Apaf-1) (158). Xanthorrhizol, a natural sesquiterpenoid, induces apoptosis via ROS-mediated p38 MAP kinase and JNK activation (159).

Although still limited, experimental evidence indicates that targeting ferroptosis represents a powerful approach to kill OSCC cells. Non-thermal plasma (NTP) causes ferroptotic cell death in OSCC cells (87). NTP is a body temperature ionized gas consisting of electrons, ions, neutral atoms, and radicals (160). Originally, NTP was found to cause oxidative stress and apoptosis in many cancer types, including OSCC (161, 162). In 2019, Sato, K. et al., demonstrated for the first time that NTP causes ferroptosis in OSCC cells through the accumulation of iron within lysosomes, the production of mitochondrial ROS, and lipid peroxidation (87). According to Sato, K. et al., the use of NTP to trigger ferroptosis in OSCC cells showed two advantages. First, the ferroptosis-mediated cytotoxic effect of NTP was less harmful to normal tissues; indeed, they found that NTP selectively killed OSCC cells without affecting the surrounding normal fibroblasts. The biological reason underlying this difference was that tumor cells are generally characterized by higher intracellular iron amount and oxidative stress compared to non-tumor cells (87, 163). Besides, due to the accessibility of the oral cavity, clinical application of NTP would be easier at this site compared to other organs (87).

In addition to chemical drugs, also natural compounds promote ferroptosis in OSCC. The natural product piperlongumine (PL), a natural alkaloid extracted from pepper, causes ferroptosis in OSCC cells (164). PL shows a cytotoxic effect in many cancer types, by causing oxidative stress, cell cycle arrest and autophagy (165, 166). According to Wang, Z.K. et al., PL triggers ferroptosis in OSCC cells by reducing the expression of SLC7A11 and GPX4 and increasing lipid peroxidation. In non-tumor cells, instead, PL seems to act as herbal medicine with very little toxicity (164).

Ferroptosis induction has also shown the potential to enhance or restore sensitivity of OSCC cells to other therapeutic strategies. As mentioned before, radiotherapy is the first line of treatment for most OSCC patients (89, 167). Radiotherapy is typically administered postoperatively and depends on variables such as the primary tumor size, positive surgical margins, and the presence of perineural, lymphatic, and vascular invasion. In addition, chemotherapy has recently become a popular adjunct treatment for locally advanced OSCC. Even though chemotherapy is not considered a curative treatment for oral carcinomas, it can be administered prior to surgery or in conjunction with irradiation before or after surgery. Adjuvant chemotherapy and radiotherapy are becoming standard remedies for advanced oral cancers. Chemotherapy and radiotherapy cause apoptosis of OSCC cells, among others, by causing an oxidative stress injury (168). However, the clinical efficiency of both therapeutic approaches is often limited by the development of chemo- or radio-resistance, determined by the dysregulation of apoptotic pathways (1, 169, 170). In 2022, Liu, J. et al. found that exposure to hyperbaric oxygen (HBO) enhances the sensitivity to radiation therapy of OSCC cells by expanding the oxidative damage caused by the sole treatment with radiotherapy (93). Previously, it has been shown that OSCC cells undergo apoptosis following HBO, perhaps via activation of the MAP kinase pathway (171). According to Liu, J. et al., the molecular basis of the synergistic effect of HBO and radiotherapy does not rely on apoptosis induction, but rather on ferroptosis. HBO, indeed, inhibits GPX4 expression and causes lipid peroxidation (93). In parallel, Han, L., et al. found that carnosic acid, a polyphenolic abietane diterpene derived from rosemary, sensitizes resistant OSCC cells to cisplatin by causing ferroptosis (91). Carnosic acid has previously shown cytotoxic effects in several cancers and to enhance the therapeutic efficacy of tamoxifen in estrogen receptor (ER) positive breast cancer (172, 173). Han, L., et al. demonstrated that carnosic acid suppresses the antioxidant pathway mediated by nuclear factor E2 related factor 2 (Nrf2/NFE2L2) and its downstream target System Xc^−^, thus leading to ROS accumulation, lipid peroxidation, and ferroptosis in cisplatin-resistant OSCC cells. Notably, in this study, carnosic acid seemed to preserve normal oral keratinocytes.

More recently, melatonin was found to synergize with erastin to trigger ferroptosis in OSCC cells by reducing the intracellular levels of GSH and increasing those of lipid ROS (97). Although generally used for its anti-inflammatory and immune system regulatory properties (174), melatonin has shown to induce apoptosis and autophagy in cancer cells (175, 176). In the study by Wang, C., et al. the combinatory treatment with erastin and melatonin markedly reduced the OSCC size *in vivo*, without obvious systemic side effects (97).

## 4 Discussion

Since its discovery in 2012, ferroptosis has emerged as an “Achille's heel” particularly in certain cancers, whose pathways related to redox homeostasis, iron and lipid metabolisms are dysregulated as part of the molecular pathogenesis. Hence, targeting this vulnerability, through the use of FINs, has provided a new potential antitumor therapeutic approach) (44–46). During the last five years, an increasing amount of compelling evidence has indicated that ferroptosis-regulatory pathways are activated or disrupted during oral carcinogenesis. Compared to tumors, such as melanoma, lung cancer, breast cancer, and colorectal cancer, whose relationship with ferroptosis has been under investigation from longer, the vast majority of studies in OSCC indicate that ferroptosis occurs mainly through the inhibition of the antioxidant pathways driven by the System Xc^−^/GSH/GPX4 axis and the reprogramming of cytoplasmic iron metabolism progression (19). Data demonstrating the contribution of tumor metabolism, such as glucose and glutamine metabolism, but also that of mitochondrial iron and of mevalonate, the role of Coenzyme Q in the protection against ferroptosis, or the role of major oncogenic signaling pathways in the regulation of ferroptosis, such as AMPK pathways and Hippo pathway, are still missing.

As the understanding of ferroptosis-regulating pathways in OSCC has caught on, new druggable targets and new ferroptosis modulators have emerged as likely to trigger oral cancer cell death. In this regard, System Xc^−^/GSH/GPX4 proteins synthesis and activity appear as the most targeted ferroptosis regulators both *in vitro* and *in vivo*, thus confirming a prominent role of this pathway in OSCC progression (33, 34, 37, 38). Few studies show that the epigenetic machinery of OSCC may have an appreciable role in the regulation of ferroptosis target genes, either through the direct activity of miRNAs or the competitive regulatory mechanism of endogenous RNA mediated by circRNAs (29, 36). Furthermore, three important information arises from the pharmacological studies assessing the effects of FINs in OSCC. First, the array of compounds able to trigger ferroptosis in OSCC is not limited to chemical drugs but also natural compounds seem to have considerable research value. Second, OSCC cells appear in some cases more sensitive to ferroptosis than their corresponding normal oral epithelial cells *in vitro*. These data highlight the existence of appropriate therapeutic windows that would allow selective ferroptosis induction in OSCC while sparing normal tissues. Third, the use of ferroptosis inducing compounds in combination with other anticancer therapies, such as chemo-radiotherapy, leads to the induction of mixed-type cell deaths that enhances tumor suppression and counteract resistance of OSCC cells to chemo-radiotherapy (87, 91, 93, 97, 164). Even in this context, ferroptosis research in OSCC lags behind other tumors, where various modalities (i.e., immunotherapy and targeted therapy), selected Food and Drug Administration-approved compounds (i.e., statins and artemisinin), and also dietary interventions (i.e., the inclusion of arachidonic acid and the restriction in methionine and cysteine) have garnered recognition for their potential in inducing or sensitizing cancer cells to ferroptosis.

Most of the factors used in clinical practice to predict prognosis in OSCC are based on clinical and histological characteristics. However, OSCC patients displaying clinical and pathological similarities may show different outcomes. Hence, clinicopathological factors alone are not sufficient to efficiently predict prognosis and exploring new prognostic models from a biological perspective appears mandatory (128). Recently, prognostic models based on ferroptosis have provided a valid tool to predict OS in different cancers. In this regard, five FRGs- or frlncRNAs- signatures have been found to correlate with prognosis of OSCC patients in silico. Interestingly, these ferroptosis-related signatures agree that the poor prognosis of OSCC patients might result from the dysregulation of immune status. In addition to providing a list of new potential prognostic biomarkers, that need to be further validated in larger cohort of patients, this finding puts the ground for future in-depth analysis of the relationship between ferroptosis and antitumor immune response in OSCC and opens up promising horizons for the application of immunotherapy in combination with FINs in this disease (89, 92, 94–96).

Ferroptosis research in cancer has shown that there is crosstalk between ferroptosis and other RCD pathways as it involves similar gene mutations and protein alterations (19). In this review, we have noticed that the vast majority of FRGs with prognostic values in OSCC are functionally involved in autophagy (Figure 4). *ATG5*, *MAP1LC3A*, *BECN1* are the main drivers of phagophore formation and maturation and are considered autophagy-related biomarkers able to predict prognosis in OSCC (177). To sustain autophagy flux, transcriptional upregulation of genes encoding for proteins involved in this process is crucial. In this regard, the transcription factors DDIT3 and DDIT4 are key players. Under hypoxic conditions, DDIT3 promotes the transcriptional upregulation of *MAP1LC3A* and *ATG5* (178). DDIT3 can also stimulate autophagosome formation through downregulation of *BCL2* expression. Indeed, when present, BCL2 binds to BECN1 and hinders the generation of VPS34 complex I and, thus, the phosphorylation of phosphatidylinositol-3 (PI3) on autophagic membranes (178). BNIP3 is an outer mitochondrial membrane (OMM) protein, belonging to the BCL2-family, that directly interacts with *MAP1LC3* to mediate mitochondrial autophagy. Under hypoxic conditions, BNIP3 is induced and localized to the mitochondria, where it triggers loss of membrane potential, increases ROS production, and causes mitophagy. BNIP3 can regulate autophagic cell death also by competing with BECN1 for binding to BCL2 (179). In OSCC, BNIP3 has been found to drive mitophagy and to sensitize OSCC cells to cisplatin treatment (180). CISD2, also known as nutrient-deprivation autophagy factor-1, binds to BCL2 at the endoplasmic reticulum (ER), contributes to the interaction of BCL2 with BECN1 thereby hindering formation of the BECN1-PIK3C3 autophagosome-initiating complex in response to nutrient stress (181). MAP3K5 promotes autophagy partly converging into DDIT3-mediated pathways. Briefly, in response to ER stress signals, the ERN1 (Endoplasmic reticulum to nucleus signaling 1) induces MAP3K5 to activate MAPK/p38 (mitogen-activated protein kinase), which in turn phosphorylate BCL2 (182). DDIT4 expression increases under the hypoxic condition and causes or sensitizes cancer cells to autophagy through the inhibition of mTOR signaling pathway (183). Notably, the mTOR pathway is altered in around 30% of oral cancer, where it modulates proliferation, invasion, angiogenesis, migration, and metabolism of OSCC cells. Hence, targeting mTOR exerts a great antitumor activity in OSCC and relative clinical trials show encouraging results (184). Besides, DDIT4 has been previously found upregulated in oral cancer and associated with a bad prognosis in terms of advanced TNM stage, higher tumor mutational burden (TMB) and low immune score and infiltrations (185, 186). CA9 is a hypoxia-inducible gene that is up-regulated in tumor cells undergoing oxygen and nutrient deprivation (187). In Hela cervical cancer cells, it has been found that treatment with CA9 inhibitors causes the overexpression *MAP1LC3A* and *BECN1* and triggers autophagy (188). mTOR is activated, among others, by the cytokine receptor FLT3. In acute myeloid leukemia cells, the gain-of-functions mutations of FLT3 promotes cell survival, at least in part, through the activation of mTOR. Notably, the use of FLT3 inhibitors, i.e., sorafenib, triggers autophagy in AML cells by blocking the mTOR signaling pathway (189, 190). The pro-apoptotic BID has never been directly observed in autophagy execution. However, a comprehensive bioinformatics analysis identified BID as belonging to an autophagy-related multigene signature correlated with overall survival in OSCC (191). CDKN2A is a tumor suppressor gene as it prevents phosphorylation of Retinoblastoma (Rb) protein and inhibits cell cycle progression and cell proliferation. CDKN2A is mutated with a loss of function and deletions in more than 80% of OSCC. Plus, low expression of CDKN2A is associated with decrease in overall survival and recurrence in OSCC patients (192). Notably, CDKN2A silencing promoted autophagy and upregulated the autophagy markers LC3II and BECN1 in endometrial cancer (193). Overall, in light of these observations, we suggest giving importance to the autophagic flux during ferroptosis execution also in OSCC and we open up a new point of reflection on new possible targetable regulatory molecules.

In conclusion, we believe that ferroptosis represents a novel piece of the intricate puzzle of oral cancer biology and also a new glimmer of hope for oral cancer treatment. Nevertheless, there are still many research gaps that need to be filled to certainly bring ferroptosis among the new promising opportunities for OSCC cancer treatment. First, the translation of therapeutic interventions targeting ferroptosis vulnerabilities into clinical applications necessitates the identification and validation of OSCC patient populations whose cancers exhibit heightened susceptibility to ferroptosis, based on insights derived from cell line and preclinical studies. These studies will be necessary to unravel the differences in ferroptosis execution in diverse OSCC subtypes, to discern whether these difference stem from variances in cellular or cancer contexts, or in mutation profiles, or in features of TME. In this last regard, efforts will be required to more in-depth understand ferroptosis function in immune cell populations. Subsequently, rigorous clinical trials are needed to assess the efficacy of FINs in OSCC patients, to establish their safety when combined with other ferroptosis–sensitizing treatments, and to assess their ability to replicate preclinical successes in overcoming therapeutic resistance. Equally important will be the development of robust biomarkers or metabolomic profiles for predicting and monitoring tumor responsiveness to FINs. The establishment of reliable tools for detecting these biomarkers also in biofluids, such as saliva, is paramount to this effort.
